# The distinct roles of myosin IIA and IIB under compression stress in nucleus pulposus cells

**DOI:** 10.1111/cpr.12987

**Published:** 2021-01-07

**Authors:** Wencan Ke, Bingjin Wang, Wenbin Hua, Yu Song, Saideng Lu, Rongjin Luo, Gaocai Li, Kun Wang, Zhiwei Liao, Qian Xiang, Shuai Li, Xinghuo Wu, Yukun Zhang, Cao Yang

**Affiliations:** ^1^ Department of Orthopaedics Union Hospital Tongji Medical College, Huazhong University of Science and Technology Wuhan China

**Keywords:** actomyosin cytoskeleton, compression stress, intervertebral disc degeneration, myosin II, RhoA/ROCK

## Abstract

**Objectives:**

Inappropriate or excessive compression applied to intervertebral disc (IVD) contributes substantially to IVD degeneration. The actomyosin system plays a leading role in responding to mechanical stimuli. In the present study, we investigated the roles of myosin II isoforms in the compression stress‐induced senescence of nucleus pulposus (NP) cells.

**Material and methods:**

Nucleus pulposus cells were exposed to 1.0 MPa compression for 0, 12, 24 or 36 hours. Immunofluorescence and co‐immunoprecipitation analysis were used to measure the interaction of myosin IIA and IIB with actin. Western blot analysis and immunofluorescence staining were used to detect nuclear expression and nuclear localization of MRTF‐A. In addition, the expression levels of p‐RhoA/RhoA, ROCK1/2 and p‐MLC/MLC were measured in human NP cells under compression stress and in degenerative IVD tissues.

**Results:**

Compression stress increased the interaction of myosin IIA and actin, while the interaction of myosin IIB and actin was reduced. The actomyosin cytoskeleton remodelling was involved in the compression stress‐induced fibrotic phenotype mediated by MRTF‐A nuclear translocation and inhibition of proliferation in NP cells. Furthermore, RhoA/ROCK1 pathway activation mediated compression stress‐induced human NP cells senescence by regulating the interaction of myosin IIA and IIB with actin.

**Conclusions:**

We for the first time investigated the regulation of actomyosin cytoskeleton in human NP cells under compression stress. It provided new insights into the development of therapy for effectively inhibiting IVD degeneration.

## Graphical abstract

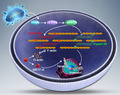

## 1 INTRODUCTION

Intervertebral disc (IVD) degeneration and secondary pathological changes are the leading cause of low back pain, which is at the top of the main reasons of chronic disability.^1^ The IVD is an avascular tissue that consists of three inter‐related structures: the highly hydrated nucleus pulposus (NP) at the centre, the highly fibrocartilaginous annulus fibrosus on the outer periphery and the endplates on the upper and lower faces.^2^ The IVD undergoes substantial mechanical stimuli during spinal motion and joint muscle movement. During loading, cells of the IVD undergo compressive, tensile, shearing deformation and fluid flows, all of which play an significant role in cell metabolism of the IVD.^3^, ^4^, ^5^, ^6^ NP cells are substantial contributors in the extracellular matrix (ECM) anabolism to maintain the gelatinous property of NP tissue, allowing it to buffer the various mechanical stimuli.^7^, ^8^ Increasingly, evidence suggests that the cell‐mediated and biological remodelling responses to mechanical stimuli play an important role in IVD degeneration.^9^, ^10^ Many studies have sought to elucidate the biological responses of NP cells to mechanical stimuli; however, the mechanical signal sensing and mechanical signal transduction processes in NP cells are still poorly understood.

The actomyosin system plays a leading role in the response to mechanical stimuli, including stretch, compression and shear forces, as well as internally generated tension and strain.^11^, ^12^ The actomyosin system consists of actin and myosin, which form stress fibres for generating contractile forces. The members of the myosin family are grouped into more than 30 classes that have different distributions and play diverse functions.^13^ Myosin II has emerged as an obvious player in a variety of force processes, such as cytokinesis, cell migration, polarization and adhesion.^14^, ^15^, ^16^ In mammalian cells, myosin II heavy chains include three different isoforms: myosin IIA, myosin IIB and myosin IIC encoded by MYH9, MYH10 and MYH14 genes, respectively. Myosin IIA and IIB can be detected in most tissues whereas myosin IIC is absent in some tissues.^17^ Although myosin IIA and IIB present a high degree of homology in the amino acid sequences of their heavy chains, they display differences in organization, intracellular distributions and molecular interactions. For example, in epithelial junction assembly, myosin IIA is parallel to the junction and provides mechanical force for the maintenance of adherens junction. Myosin IIB localizes at junctional membranes and organizes the junctional branched actin meshwork.^18^ A recent study demonstrated that myosin IIA is dedicated to generating cortex tension for faster cleavage furrow ingression in cell division, while myosin IIB acts as a stabilizing motor by reducing cortex tension.^19^ In our previous studies, we have demonstrated that compression stress can induce apoptosis of NP cells and ECM degradation during IVD degeneration.^20^, ^21^ However, little is known about the distinct roles of myosin II isoforms in compression stress‐induced senescence of NP cells.

Furthermore, the actomyosin cytoskeleton is highly regulated by the RhoA/ROCK signalling pathway. RhoA belongs to the Ras homology proteins family of 22 small GTPases.^22^ RhoA can switch between an inactive GDP‐bound form and an active GTP‐bound state, located in the cell membrane when it is active.^23^, ^24^ Its major biological functions include the generation of actomyosin bundles, stress fibres, focal adhesions and lamellipodia.^25^ Rho‐associated coiled‐coil–containing protein kinases (ROCK) are dominating regulators of the actin cytoskeleton, which downstream of the small GTPase RhoA. The ROCK family includes two members, ROCK1 and ROCK2, which have 65% homology in amino acid sequence.^26^, ^27^ The ROCK proteins act on target substrates including the myosin regulatory light chains (MLC) and a myosin‐binding subunit of the myosin phosphatase through which they regulate myosin contractility, and the LIM kinases through which they control actin depolymerization. The RhoA/ROCK signalling has been reported to act as a prominent player in responding to mechanical stimuli.^26^, ^28^, ^29^ However, the correlation between myosin II and the RhoA/ROCK signalling under compression stress has rarely been studied.

In our study, we investigated the role of myosin II subunits in compression stress‐induced senescence of NP cells. We demonstrated that compression stress differentially regulated the interaction of myosin IIA and IIB with actin. The actomyosin cytoskeleton remodelling was involved in the compression stress‐induced fibrotic phenotype mediated by MRTF‐A nuclear translocation and inhibition of proliferation in human NP cells. Moreover, our results indicated that RhoA/ROCK1 pathway activation mediated compression stress‐induced human NP cells senescence by regulating the interaction of myosin IIA and IIB with actin. Thus, we for the first time systematically investigated the regulation of actomyosin cytoskeleton in human NP cells under compression stress. It provided new insights into the development of therapy for effectively inhibiting IVD degeneration.

## 2 MATERIALS AND METHODS

### 2.1 NP tissues collection

The experimental protocols conducted in this study were approved by the Ethics Committee of Tongji Medical College, Huazhong University of Science and Technology. Pfirrmann magnetic resonance imaging‐grading system was used to evaluate the level of IVD degeneration.^30^ The control IVD tissues were collected from eight patients with idiopathic scoliosis (four males and four females; mean age 16.5 years; range 14‐18 years; grades I‐II). Degenerative IVD tissues were harvested from ten lumbar disc herniation patients (five males and five females; mean age: 38.2 years; range: 28‐59 years; grades III‐V) undergoing discectomy surgery.

### 2.2 NP cells isolation and culture

The human NP cells were separated and isolated from the eight patients with idiopathic scoliosis as described in previous work.^31^ Briefly, NP tissues were cut into small fragments and enzymatically digested in 0.2% type II collagenase and 0.25% trypsin for 3 hours. Then, the cells were cultured in Dulbecco's modified Eagle's medium (DMEM)/F12 (Gibco) containing 15% foetal bovine serum (FBS; Gibco) and 1% penicillin‐streptomycin and cultured at 37°C in a humidified atmosphere with 5% CO_2_. Cells from the second passage were identified using fluorescently labelled antibody for NP cell markers (CD24, ab31622; KRT18, ab215839; Abcam) and used in further experiments. For inhibition experiments, NP cells were either pre‐treated with CCG1423 (20 μmol/L; Sigma‐Aldrich) or Y27632 (20 μmol/L; MilliporeSigma) for 2 hours. To knock down myosin IIA and IIB, cells were transfected for 48 hours with 50 nmol/L small‐interfering RNA (siRNA) against myosin IIA, 50 nmol/L siRNA against myosin IIB (RiboBio) using Lipofectamine 2000 (Invitrogen) and immediately stimulated with 1.0 MPa compression.

### 2.3 Compression treatment

According to the previous studies, a pressure of 1.0 MPa is widely used to induce IVD degeneration.^20^, ^21^, ^32^ A compression apparatus contains a mixture of 5% CO_2_ and 95% compressed air was used to provide 1.0 MPa compression as described in our previous work.^21^ In addition, the apparatus was placed in an incubator at 37°C. NP cells were exposed to 1.0 MPa compression for 0, 12, 24 or 36 hours.

### 2.4 Total and nuclear‐cytosol protein extraction and Western blot analysis

Total protein was extracted from human NP cells by RIPA lysis buffer containing protease inhibitor (Beyotime). Nuclear and cytoplasmic proteins were extracted from NP cells by a Nuclear‐Cytosol Extraction Kit (Solarbio) according to the manufacturer's instructions. Western blot procedure was performed as previously described.^33^ Primary antibodies against the following proteins were used: myosin IIA (Abcam, ab138498, 1:1000), myosin IIB (Abcam, ab230823, 1:1000), p21 (CST, 2947, 1:1000), p53 (Proteintech, 10442‐1‐AP, 1:1000), Cyclin D1 (Abcam, ab134175, 1:10 000), CDK4 (Abcam, ab108357, 1:1000), MRTF‐A (Abcam, ab115319, 1:1000), RhoA (Abcam, ab187027,1:1000), p‐RhoA‐Ser188 (Abcam, ab41435, 1:1000), ROCK1 (Proteintech, 21850‐1‐AP, 1:1000), ROCK2 (Proteintech, 20248‐1‐AP, 1:1000), MLC (CST, 3671, 1:1000), p‐MLC‐Ser19 (CST, 3671, 1:1000), GAPDH (Affinity, AF7021, 1:2000), Lamin B (Proteintech, 12987‐1‐AP, 1:1000). GAPDH and Lamin B were used for normalization. The experiments were repeated three times.

### 2.5 Quantitative real‐time polymerase chain reaction (qRT‐PCR)

Total RNA was extracted from human NP cells with TRIzol reagent (Invitrogen), reverse‐transcribed and amplified by qRT‐PCR according to manufacturer's instructions. The primers used for qRT‐PCR were as follows:

Homo MMP3, forward 5′‐TCCTGATGTTGGTGGCTTCAG‐3′,

reverse 5′‐TGTCTTGGCAAATCCGGTGTA‐3′.

Homo MMP13, forward 5′‐TGATGGACCTTCTGGTCTTCTGG‐3′,

reverse 5′‐CATCCACATGGTTGGGAAGTTCT‐3′.

Homo ADAMTS5, forward 5′‐GCCATTGTAATAACCCTGCACC‐3′,

reverse 5′‐TCAGTCCCATCCGTAACCTTTG‐3′.

Homo COL1A1, forward 5′‐GGGCAAGACAGTGATTGAATACA‐3′,

reverse 5′‐ GGATGGAGGGAGTTTACAGGAA‐3′.

Homo COL2A1, forward 5′‐CACACTGGTAAGTGGGGCAAGACCG‐3′, reverse 5′‐GGATTGTGTTGTTTCAGGGTTCGGCT‐3′.

Homo aggrecan, forward 5′‐GAAGACGACATCACCATCCCAG‐3′,

reverse 5′‐CTGTCTTTGTCACCCACACATG‐3′.

Homo GAPDH, forward 5′‐TCAAGAAGGTGGTGAAGCAGG‐3′,

reverse 5′‐TCAAAGGTGGAGGA GTGGGT‐3′. GAPDH was used for control.

### 2.6 Senescence‐associated β‐galactosidase (SA‐β‐gal) staining

The senescence of NP cells was determined using a SA‐β‐gal Staining Kit (Beyotime) following the manufacturer's protocol. Briefly, cells were washed with PBS (pH 7.4) and fixed in an SA‐β‐gal working solution (pH 6.0) at 37°C without CO_2_ overnight. Then, the average percentage of total SA‐β‐gal‐positive cells was calculated for quantitative analysis.

### 2.7 Immunofluorescence analysis

Immunofluorescence analysis was performed as previously described.^33^ Briefly, 4% paraformaldehyde was used to fix attached human NP cells, then 0.2% Triton X‐100 in PBS was used to permeabilize. The slides were washed in PBS and blocked with 2% bovine serum albumin (BSA) in PBS for 2 hours at 37°C and then incubated with primary antibodies against: myosin IIA (Abcam, ab138498, 1:200), myosin IIB (Abcam, ab230823, 1:100), MRTF‐A (Abcam, ab115319, 1:200). After washed twice, the slides were subsequently treated with secondary goat anti‐rabbit antibody (Boster) at 37°C for 2 hours. Nuclei were co‐stained for 5 minutes with 0.1 g/mL DAPI (Beyotime, Nantong, China), and images were captured under a microscope (Olympus, BX53). To analyse the nuclear‐to‐cytoplasmic fluorescent intensity ratio of MRTF, the nuclear intensity and cytoplasmic intensity of 20 cells from each group were calculated and analysed by Image J (NIH). For co‐localization analysis of myosin II isoforms with F‐actin, the Pearson coefficient was calculated by Image J (NIH) and the Coloc 2 plugin.

### 2.8 RNA interference

Knockdown of myosin IIA or IIB in NP cells were realized by transfected with short interfering RNA (siRNA). Si‐RNA against myosin IIA (si‐ myosin IIA), myosin IIB (si‐ myosin IIB) and scrambled siRNA (si‐NC) were synthesized by RiboBio (Guangzhou, China) and transfected with Lipofectamine 2000 (Invitrogen) according to the standard protocol. Three different siRNAs were designed for each molecule and the target sequences were as follows: myosin IIA‐1, 5′‐GCATCAACTTTGATGTCAA‐3′; myosin IIA‐2, 5′‐GCAACACGGAGCTGATCAA‐3′; myosin IIA‐3, 5′‐ACAAGAACCTGCCCATCAT −3′; myosin IIB‐1, 5′‐CCAGAAGAGTGACAATGCA‐3′; myosin IIB‐2, 5′‐GACCCAAACTTGTACAGAA‐3′; myosin IIB‐3, 5′‐GAAGGATCGCTACTATTCA −3′.

### 2.9 Flow cytometric and cell cycle analysis

The human NP cells were harvested through trypsinization, fixed with 70% cold ethanol overnight at 4°C. Next, the cells were incubated with RNase (50 μg/ml; KeyGEN) for 30 min at 37°C, followed by propidium iodide dye (50 μg/ml; KeyGEN) for a further 30 min. The cells were then analysed using flow cytometry (BD FACSCalibur; BD Biosciences).

### 2.10 Immunoprecipitation

The Protein A/G Magnetic Beads (MCE) was washed with lysis buffer three times before mixing with antibody. Prior to immunoprecipitation, 5 μg of purified antibodies against myosin IIA, myosin IIB, actin filaments or normal IgG were mixed with 400 μl of lysis buffer with 25 μl beads and incubated 2 hours at 4°C on a rocker table. The prepared antibody‐beads complex was added to 1 ml of cell lysates (1 mg/ml) and incubated with rocking for 4 hours at 4°C. Then, magnetic separation was performed, and the supernatant was removed. Subsequently, the immune complex was mixed with 5 × loading buffer and then boiled for 10 minutes before the analysis by Western blot.

### 2.11 Immunohistochemistry

NP samples were fixed in 10% formaldehyde for 24 hours and embedded in paraffin. Then, the samples were sliced into 4‐μm sections. Immunohistochemistry was carried out as described previously.^34^ The sections were incubated with antibodies against: p‐RhoA‐Ser188 (Abcam, ab41435, 1:100), ROCK1 (Proteintech, 21850‐1‐AP, 1:100), ROCK2 (Proteintech, 20248‐1‐AP, 1:50), p‐MLC‐Ser19 (CST, 3671, 1:100). Staining was performed using the Dako REAL EnVision Detection System, Peroxidase/DAB+, Rabbit/Mouse (Dako Cytomation), according to the manufacturer's instructions. The sections were imaged by microscopy (Olympus).

### 2.12 Statistical analysis

Data are presented as the means ± SD of three independent experiments. Statistical analyses were performed using GraphPad Prism 8.0 software. Differences between groups were evaluated with Student's t test or one‐way ANOVA by analysis of variance. *P*‐value < .05 was considered statistically significant.

## 3 RESULTS

### 3.1 Compression stress induced the senescence of human NP cells

**FIGURE 1:**
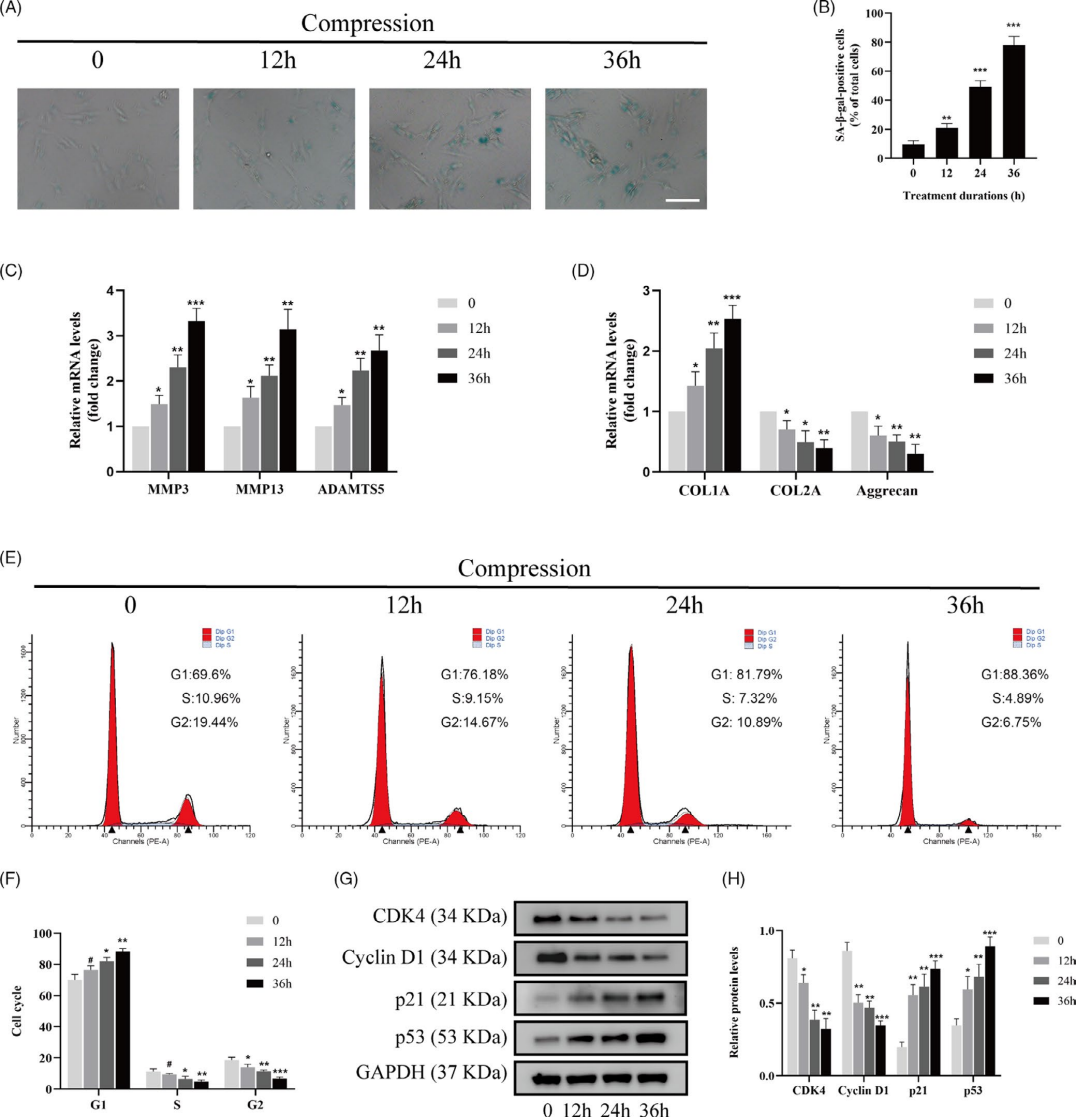
Compression stress induced the senescence of human NP cells. The human NP cells were cultured under 1.0 MPa compression for 0, 12, 24 and 36 h. (A, B) The level of senescent cells in different time was assessed by SA‐β‐gal staining. Scale bar: 50 μm. (C, D) mRNA levels of the ECM remodelling proteinases (MMP3, MMP13 and ADAMTS5) and ECM component (COL1A, COL2A and aggrecan) were quantified using qRT‐PCR. (E, F) The proportions of cells in each cycle were measured through flow cytometry with different time of compression stress. (G, H) CDK4, Cyclin D1, p21 and p53 expression levels in human NP cells subjected to different compression time were measured through Western blot analysis and normalized to that of GAPDH. Data were presented as the mean ± SD (n = 3). #No significance, vs control; *P < .05, vs control; **P < .01, vs control; ***P < .001, vs control

To investigate the senescence level of human NP cells under compression stress, we used SA‐β‐gal staining to access cellular senescence. In addition, changes in ECM metabolism and cell proliferation were measured. As shown in Figure 1A, B, the percentage of SA‐gal‐positive cells strongly increased with time from 0 to 36 hours. Furthermore, 1.0 MPa compression increased the expression of ECM remodelling proteinases (MMP3, MMP13 and ADAMTS5) over time (Figure 1C). As the exposure to compression stress was prolonged, there was a progressive reduction in the expression of aggrecan and collagen type II genes (Figure 1D). Simultaneously, collagen type I expression increased, indicating a fibrotic phenotype in human NP cells. In addition, flow cytometric and cell cycle analysis were used to determine the proliferation of human NP cells. As shown in Figure 1E, F, compression stress promoted more cells to transit to the G1 phase and decreased the percentage of the cells arrested in the S phase. To further validate the role of compression stress on the proliferation of human NP cells, protein levels of CDK4, cyclin D1, p21 and p53 were measured via Western blot analyses, respectively (Figure 1G, H). The expression levels of CDK4 and cyclin D1 notably decreased, while the protein levels of p21 and p53 increased significantly after 36 hours. These results suggested that compression stress accelerated cell catabolism and inhibit the proliferation of human NP cells.

### 3.2 Compression stress regulated the interaction of myosin IIA and IIB with actin

**FIGURE 2:**
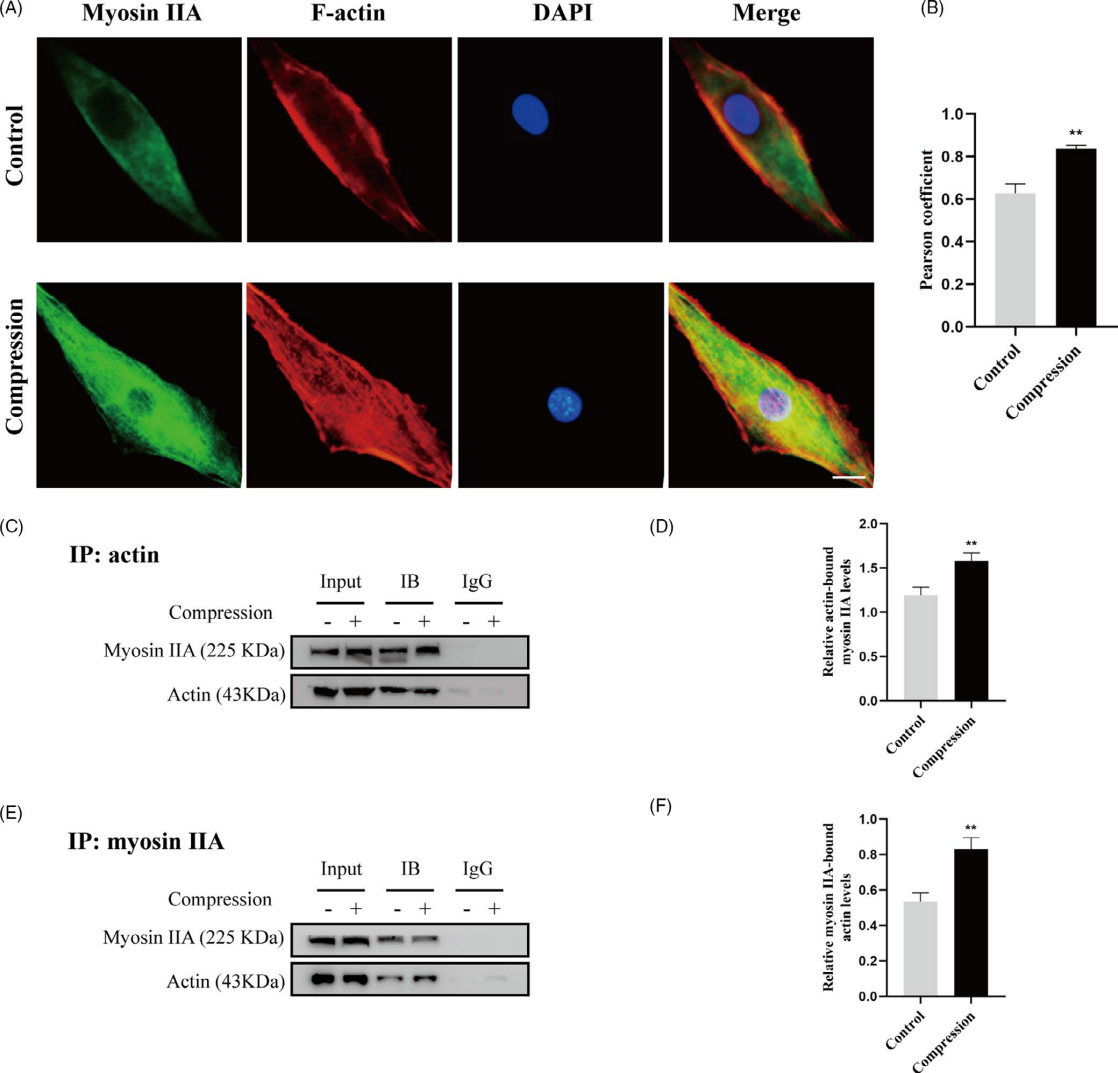
Compression stress increased the interaction of myosin IIA with actin. (A) Human NP cells untreated or treated with compression stress for 36 h were stained with myosin IIA (green), F‐actin (red) and DAPI (blue). Scale bar: 5 μm. (B) The co‐localization of myosin IIA with F‐actin was evaluated on the basis of Pearson coefficients. (C, D) Protein interaction between myosin IIA and actin was determined by co‐immunoprecipitation. Following treatment, cell lysates were immunoprecipitated with anti‐actin antibody. Isotype‐matched (IgG) served as negative control. Each precipitated sample was detected for the presence of myosin IIA and actin by immunoblot analysis using specific antibodies. Whole cell lysates prior to the immunoprecipitation served as input controls. (E, F) Cell lysates were immunoprecipitated with anti‐non‐muscle myosin IIA antibody, the next steps are described above. Data were presented as the mean ± SD (n = 3). **P < .05, vs control

**FIGURE 3:**
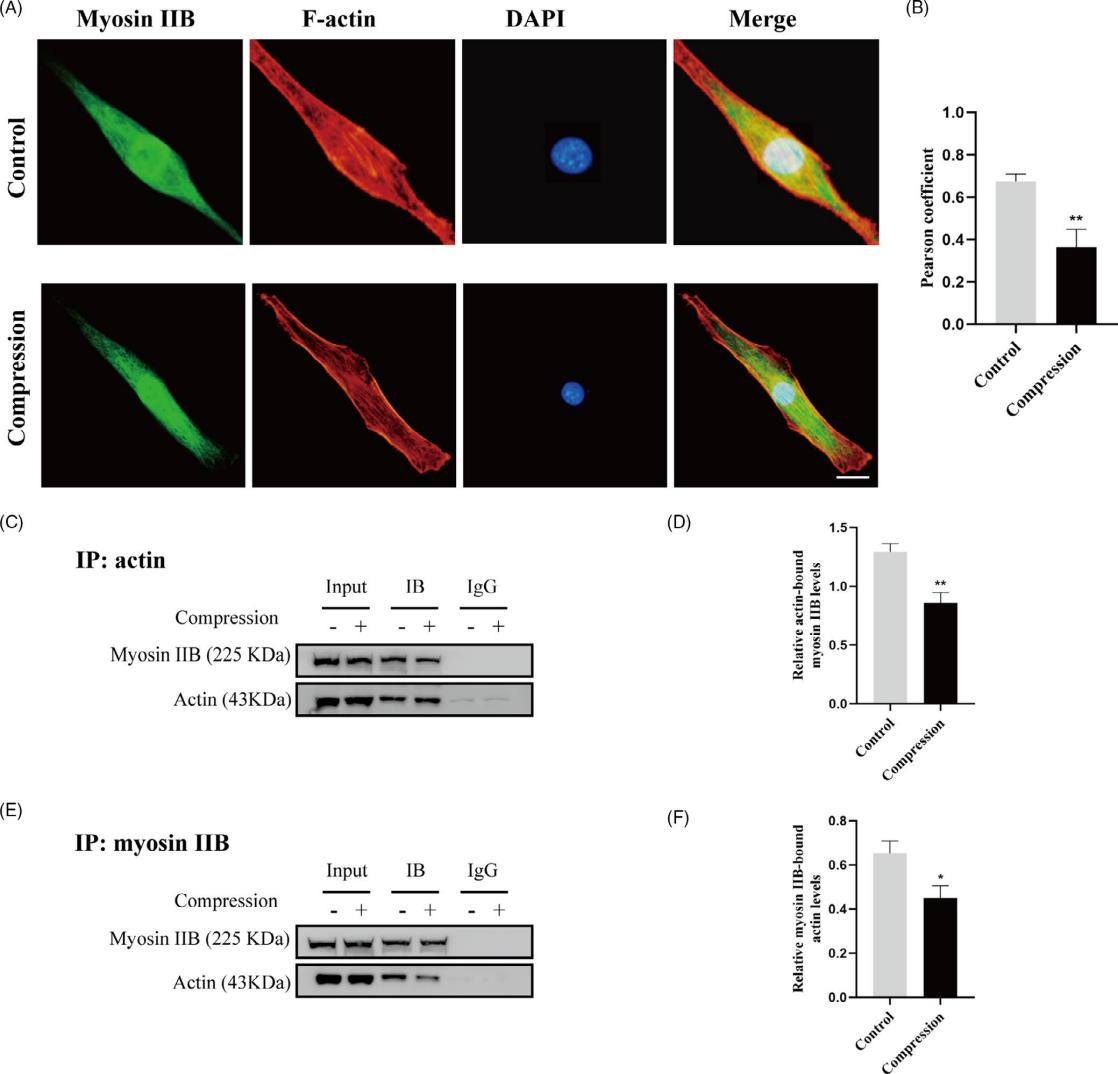
Compression stress decreased the interaction of myosin IIB with actin. (A) Human NP cells untreated or treated with compression stress for 36 h were stained with myosin IIB (green), F‐actin (red) and DAPI (blue). Scale bar: 5 μm. (B) The co‐localization of myosin IIB with F‐actin was evaluated on the basis of Pearson coefficients. (C, D) Protein interaction between myosin IIB and actin was determined by co‐immunoprecipitation. Following treatment, cell lysates were immunoprecipitated with anti‐actin antibody. Isotype‐matched (IgG) served as negative control. Each precipitated sample was detected for the presence of myosin IIB and actin by immunoblot analysis using specific antibodies. Whole cell lysates prior to the immunoprecipitation served as input controls. (E, F) Cell lysates were immunoprecipitated with anti‐non‐muscle myosin IIB antibody, the next steps are described above. Data were presented as the mean ± SD (n = 3). *P < .05, vs control; **P < .01, vs control

Given that the actomyosin system acts as a prominent player in responding to mechanical stimuli, it is important to explore how it assembles into the necessary structures in human NP cells under compression stress. To understand the role of myosin IIA and IIB in the compression stress‐induced senescence of NP cells, the interaction of myosin II subunits and F‐actin under compression stress were measured. In normal human NP cells, myosin IIA tended to be broadly peripheral (Figure 2A), while myosin IIB was distributed throughout the cytoplasm (Figure 3A). Compression stress exposure induced dramatic changes in the reorganization of myosin IIA and IIB with F‐actin. Immunofluorescence analysis revealed that myosin IIA exhibited an increased co‐localization with F‐actin exposed to compression than normal (Figure 2A). Quantitative analysis by Pearson coefficient indicated that myosin IIA and F‐actin showed significant co‐localization under compression (Figure 2B). Co‐immunoprecipitation analysis also confirmed the increased interaction of myosin IIA and F‐actin induced by compression stress (Figure 2C‐F). In contrast, immunofluorescence and co‐immunoprecipitation analysis both demonstrated that compression stress reduced the interaction of myosin IIB and F‐actin (Figure 3A‐F). Overall, these findings indicated that compression stress differentially regulated the interaction of myosin IIA and IIB with actin.

### 3.3 Compression stress induced the nuclear translocation of MRTF‐A

**FIGURE 4:**
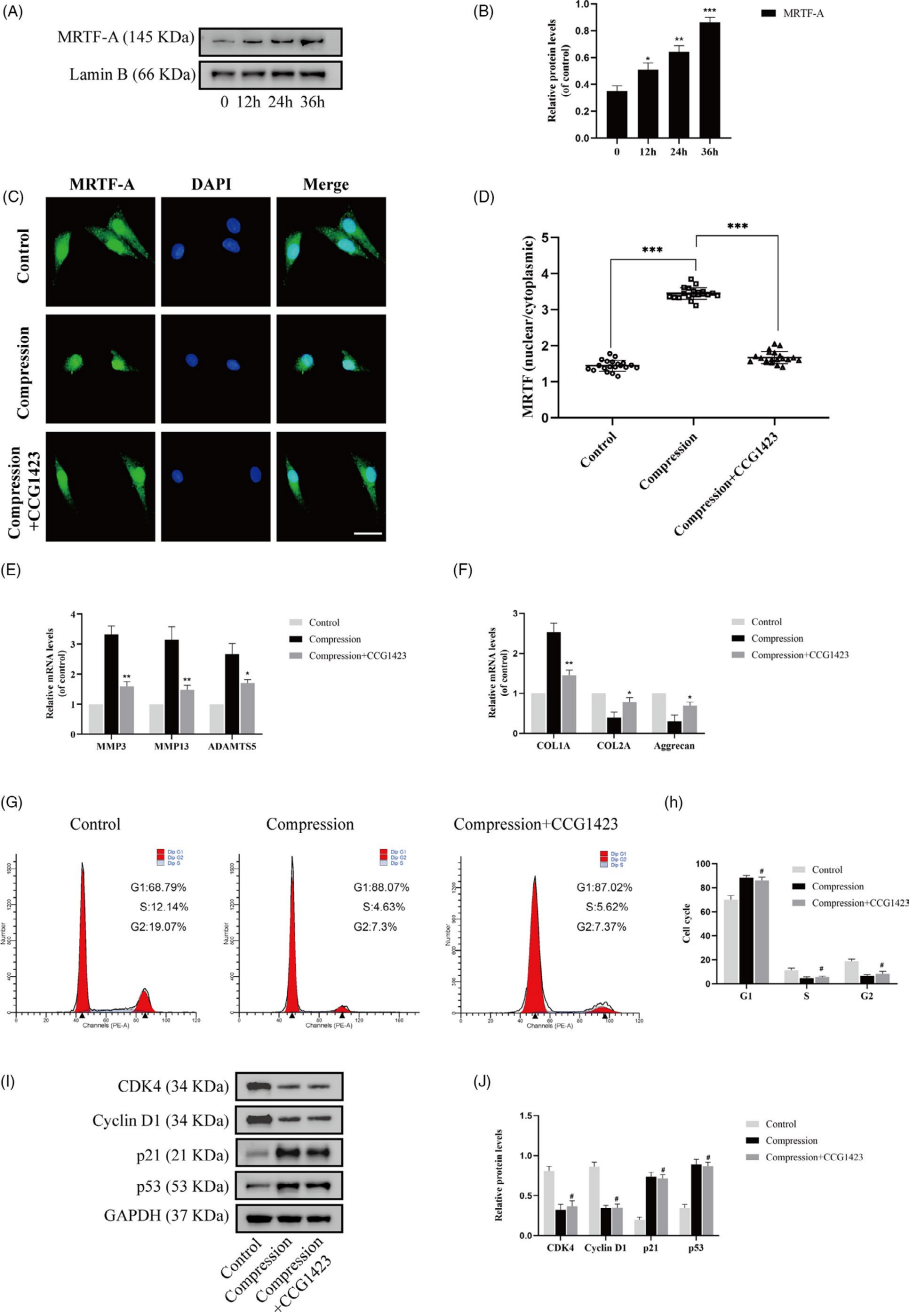
Compression stress induced the nuclear translocation of MRTF‐A. (A, B) Nuclear expression levels of MRTF‐A in human NP cells subjected to different compression time measured through Western blot analysis and normalized to that of Lamin B. Data were presented as the mean ± SD (n = 3). *P < .05, vs control; **P < .01, vs control; ***P < .001, vs control. (C, D) Human NP cells untreated or pretreated with 20 μM CCG1423 for 2 h prior to compression stress for 36 h were stained with MRTF‐A (green) and DAPI (blue) (n = 20). Scale bar: 10 μm. (E, F) mRNA levels of the ECM remodelling proteinases (MMP3, MMP13 and ADAMTS5) and ECM component (COL1A, COL2A and aggrecan) were measured after the human NP cells were treated with CCG1423. (G, H) The proportions of cells in each cycle were measured through flow cytometry after the human NP cells were treated with CCG1423. (I, J) CDK4, Cyclin D1, p21 and p53 expression levels in different groups were measured through Western blot analysis and normalized to that of GAPDH. Data were presented as the mean ± SD (n = 3). #No significance, vs compression group; *P < .05, vs compression group; **P < .01, vs compression group

Since actin often aggregates into stress fibres to perform specific functions, we hypothesized that compression stress might stimulate the formation of F‐actin by recruiting globular actin (G‐actin) molecules, resulting in the release of MRTF‐A (myocardin‐related transcription factor A) from its complex with G‐actin. Once free in the cytoplasm, MRTF‐A is translocated into the nucleus where it associates with serum‐response factor (SRF) to activate the transcription of target genes, notably fibroblast‐like matrix genes. As shown in Figure 4A‐D, Western blot analysis and immunofluorescence staining confirmed upregulated nuclear expression and nuclear localization of MRTF‐A in human NP cells under compression stress. To explore the relationship between the nuclear translocation of MRTF‐A and cell metabolism and proliferation of human NP cells, CCG1423 was used to inhibit MRTF‐A nuclear translocation under compression stress. Our results revealed that CCG1423 protects human NP cells against catabolism and it preserves their primary phenotype (Figure 4E, F). However, the inhibition of MRTF‐A nuclear translocation did not rescue the compression stress‐induced decrease in proliferation of human NP cells (Figure 4G‐J). These results indicated that the nuclear translocation of MRTF‐A mediated a fibrotic phenotype in human NP cells under compression stress, while the inhibition of proliferation was independent of MRTF‐A.

### 3.4 Distinct effects of myosin IIA or IIB knockdown in NP cells under compression stress

**FIGURE 5:**
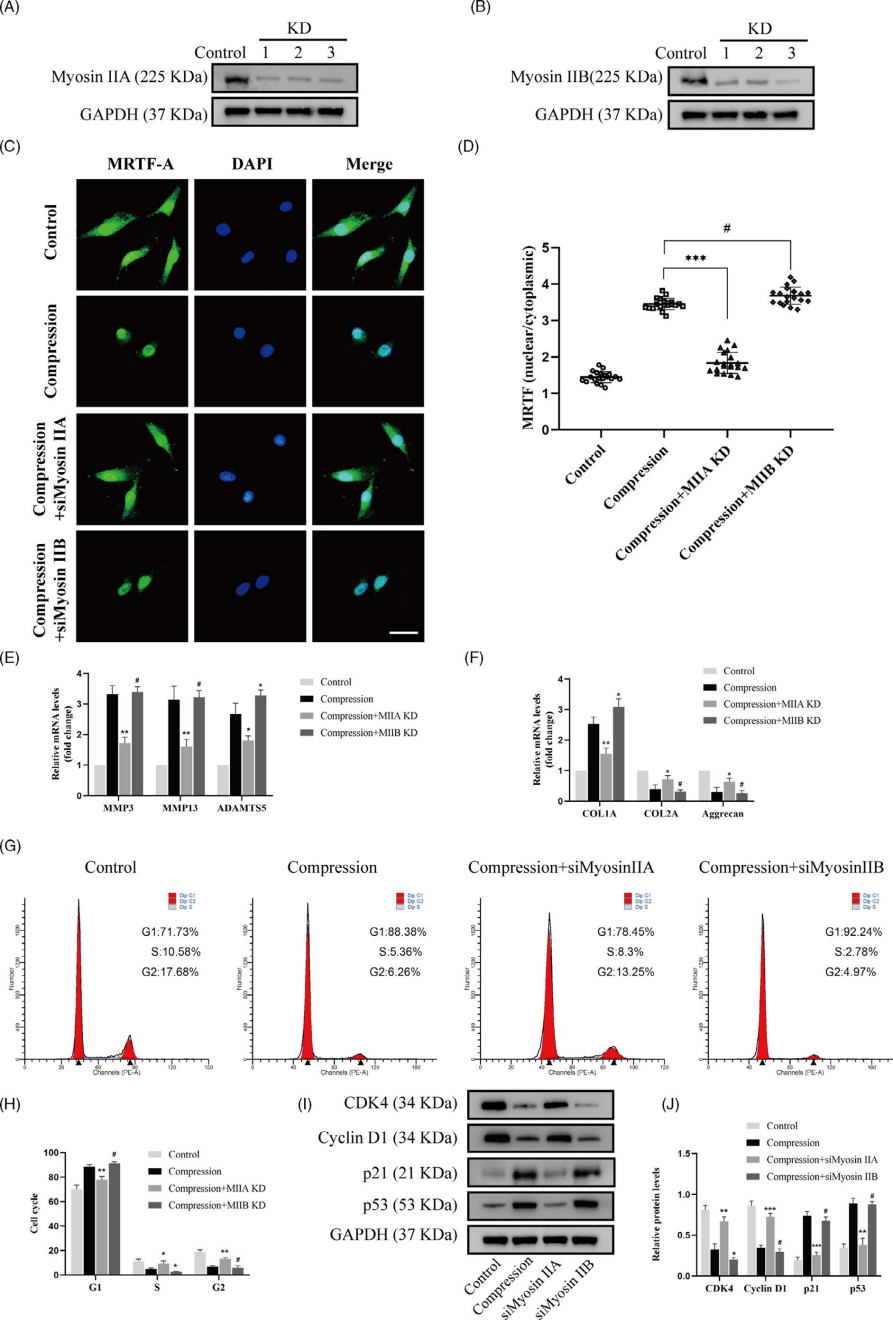
Distinct effects of myosin IIA or IIB knockdown on compression stress‐induced human NP cells senescence. NP cells were transfected with siRNAs against myosin IIA or myosin IIB as described in experimental procedures. (A, B) Myosin IIA and IIB knockdown efficiency was shown by Western blot. (C, D) Human NP cells in different groups were stained with MRTF‐A (green) and DAPI (blue) (n = 20). Scale bar: 10 μm. (E, F) mRNA levels of the ECM remodelling proteinases (MMP3, MMP13 and ADAMTS5) and ECM component (COL1A, COL2A and aggrecan) were measured after the human NP cells were treated with siRNAs against myosin IIA or myosin IIB. (G, H) The proportions of cells in each cycle were measured through flow cytometry after the human NP cells were treated with siRNAs against myosin IIA or myosin IIB. (I, J) CDK4, Cyclin D1, p21 and p53 expression levels in different groups were measured through Western blot analysis and normalized to that of GAPDH. Data were presented as the mean ± SD (n = 3). #No significance, vs compression group; *P < .05, vs compression group; **P < .01, vs compression group; ***P < .001, vs compression group

To determine the specific roles of myosin IIA and IIB in human NP cells under compression stress, knockdown of myosin IIA or IIB was achieved by transfection with siRNA (siMyosin IIA or siMyosin IIB). Three different siRNAs were designed for each molecule, and the inhibitory efficiency in human NP cells was analysed. Western blot analyses revealed that all siRNA achieved high silencing efficiency (Figure 5A, B). The NP cells were transfected with siRNA with the most effective inhibitory efficiency (siMyosin IIA‐1 or siMyosin IIB‐3) and were then used in following treatment group. Interestingly, immunofluorescence analysis indicated that knockdown of myosin IIA substantially inhibited the compression stress‐induced nuclear localization of MRTF‐A in human NP cells (Figure 5C, D). On the contrary, knockdown of myosin IIB induced a minor increase in MRTF‐A nuclear translocation under compression stress. As expected, inhibition of myosin IIA reduced ECM remodelling proteinases in human NP cells and preserved primary phenotype (Figure 5E, F). However, inhibition of myosin IIB increased the transcriptional levels of ADAMTS5 and collagen type I. In addition, flow cytometric analysis showed that myosin IIA knockdown resulted in a decrease in the G1 phase and an increase in the S and G2 phases in human NP cells, while myosin IIB knockdown promoted more cells to transit to the S phase (Figure 5G, H). Moreover, the cycle‐associated proteins levels also confirmed the same trend. Inhibition of myosin IIA resulted in the upregulation of CDK4 and cyclin D1 and the downregulation of p21 and p53, while inhibition of myosin IIB resulted in a more severe downregulation of CDK4 (Figure 5I, J). Our results revealed the diverse effects of myosin IIA or IIB knockdown in NP cells under compression stress.

### 3.5 Compression stress induced RhoA/ROCK1 pathway activation in human NP cells

**FIGURE 6:**
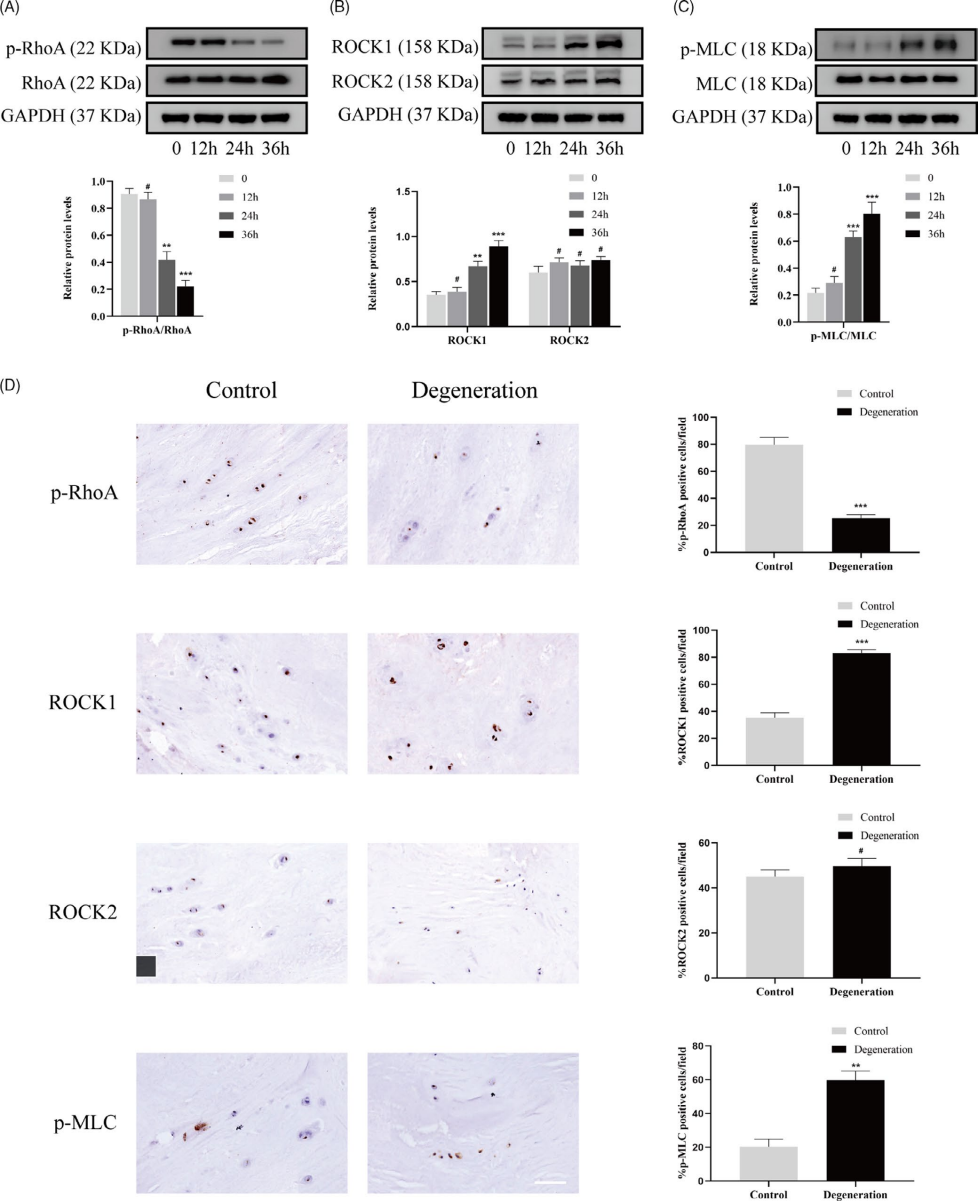
Compression stress induced RhoA/ROCK1 pathway activation in human NP cells. The protein expression level of p‐RhoA and RhoA (A), ROCK1 and ROCK2 (B), p‐MLC and MLC (C) subjected to different compression time were measured by Western blot and analysed statistically in different groups. GAPDH was used as control. (D) Immunohistochemistry analysis of p‐RhoA, ROCK1, ROCK2 and p‐MLC in control and degeneration groups. Scale bar: 50 μm. Data were presented as the mean ± SD (n = 3). #No significance, vs control; *P < .05, vs control; **P < .01, vs control; ***P < .001, vs control

Given that the actomyosin cytoskeleton is highly regulated by the RhoA/ROCK signalling pathway, we hypothesized that this pathway was activated in human NP cells under compression stress. Western blot analysis demonstrated that compression stress increased the level of phosphorylated RhoA (p‐RhoA, a marker of the inactive form of RhoA) with time from 0 to 36 hours, while the expression of RhoA did not change (Figure 6A). In addition, the expression of ROCK1 increased progressively as the exposure to compression stress was prolonged (Figure 6B). In contrast, compression stress did not affect the protein level of ROCK2. MLC phosphorylation (p‐MLC, a marker of the active form of MLC) increased after 24 hours of compression stress and the ratio of p‐MLC to MLC increased with time (Figure 6C). Furthermore, immunohistochemistry showed an increase in the proportion of p‐RhoA‐, ROCK1‐ and p‐MLC‐positive cells in the IVD degeneration group compared to that in the control group, while no obvious change was detected in ROCK2 expression (Figure 6D). Together, these findings indicated that compression stress induced RhoA/ROCK1/p‐MLC pathway activation in human NP cells.

### 3.6 Inhibition of the RhoA/ROCK1 pathway attenuated compression stress‐induced human NP cells senescence by regulating the actomyosin cytoskeleton remodelling

**FIGURE 7:**
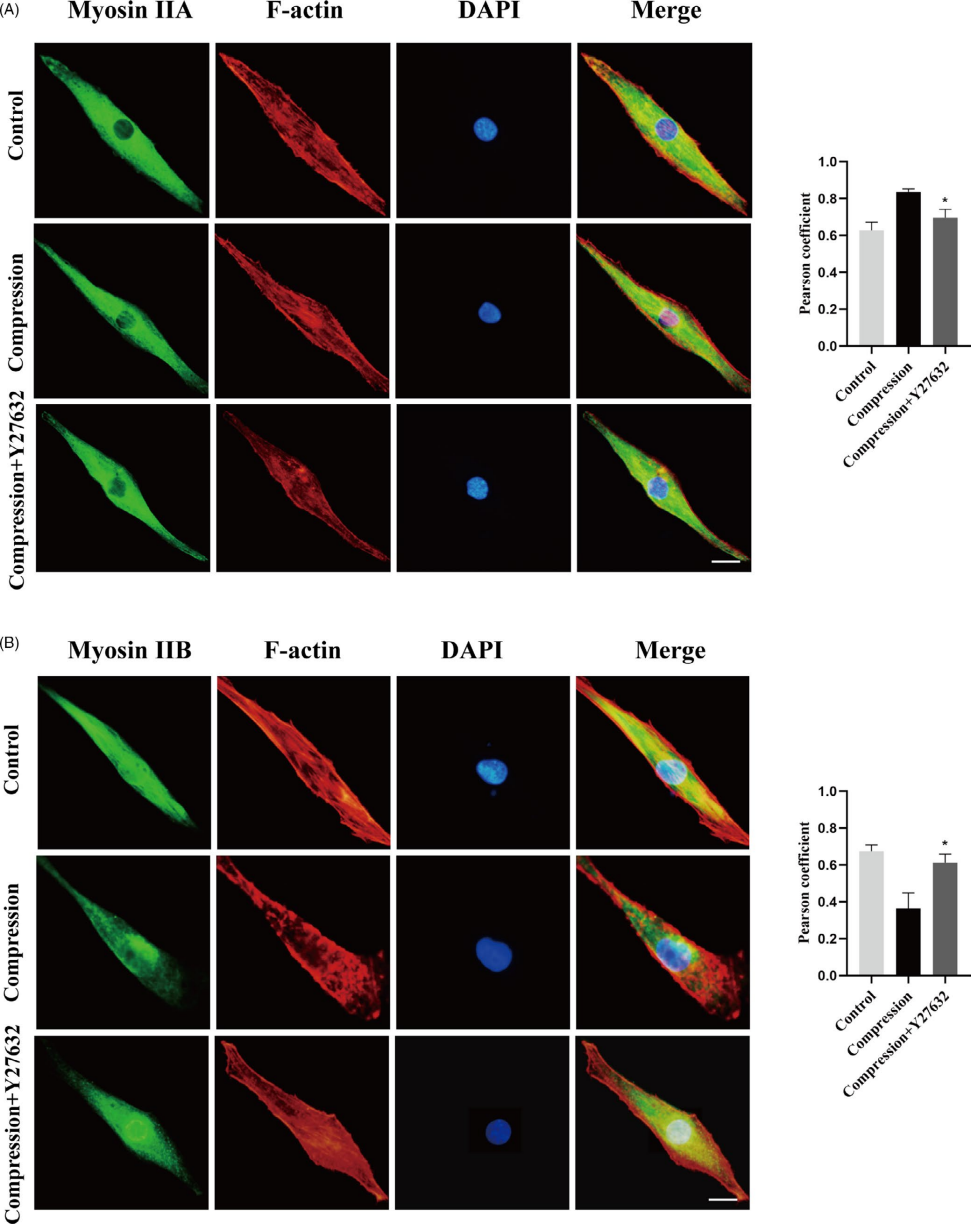
RhoA/ROCK1 pathway regulated the interaction of myosin IIA and IIB with actin in human NP cells. (A, B) Human NP cells untreated or pretreated with 20 μM Y27632 for 2 h prior to compression stress for 36 h were stained with myosin IIA or IIB (green), F‐actin (red) and DAPI (blue). Scale bar: 5 μm. The co‐localization of myosin IIA or IIB with F‐actin was evaluated on the basis of Pearson coefficients. Data were presented as the mean ± SD (n = 3). *P < .05, vs compression

To explore the role of the RhoA/ROCK1 pathway in compression stress‐induced human NP cells senescence, Y27632, a ROCK1 inhibitor, was used to inhibit the RhoA/ROCK1 pathway. Interestingly, immunofluorescence and statistical analysis revealed that Y27632 significantly reversed the co‐localization between myosin IIA and actin filaments (Figure 7A). In addition, inhibition of the RhoA/ROCK1 pathway rescued the decreased interaction between myosin IIB and actin under compression stress (Figure 7B).

**FIGURE 8:**
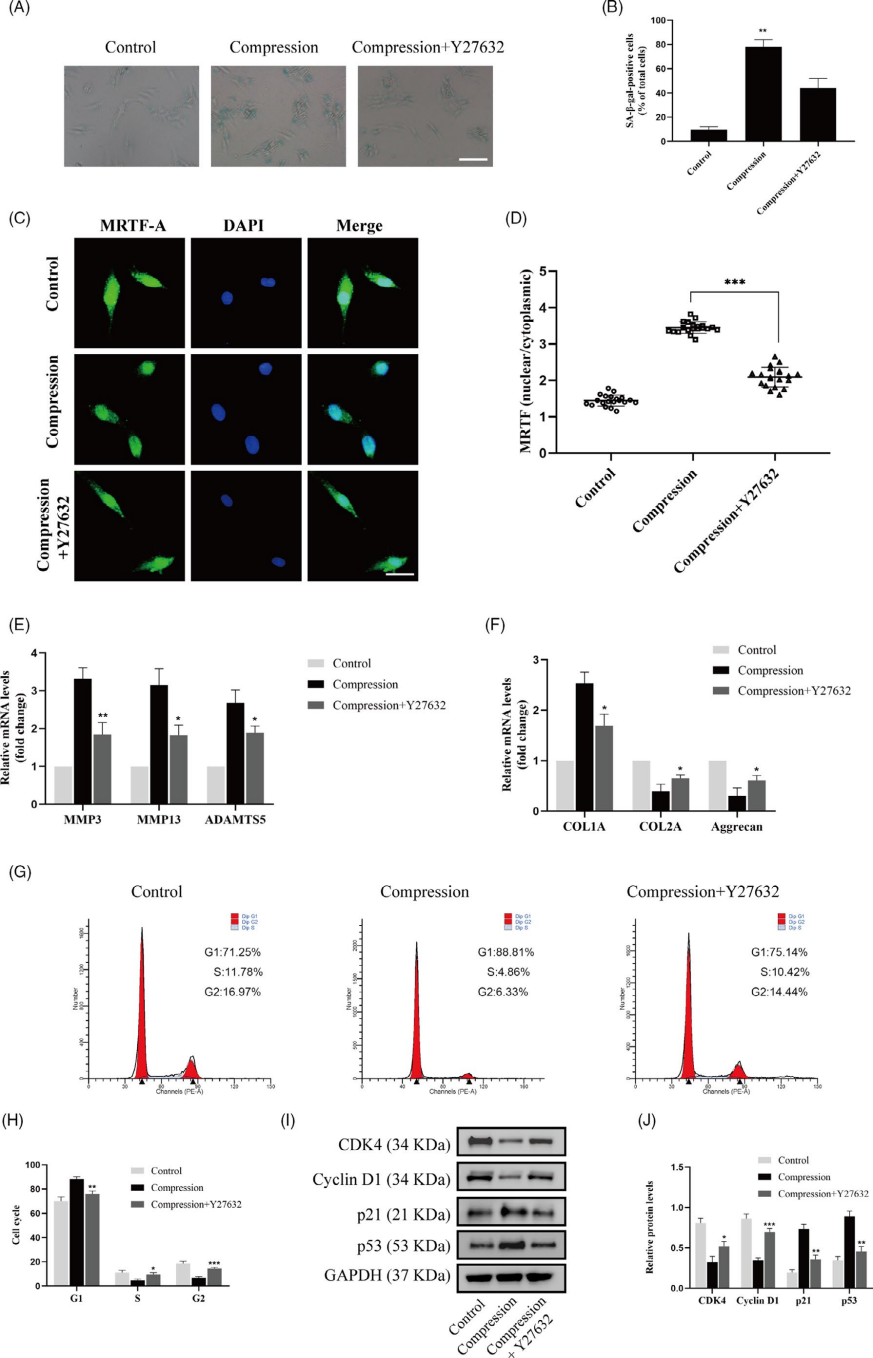
Inhibition of RhoA/ROCK1 pathway attenuated compression stress‐induced human NP cells senescence. (A, B) The level of senescent cells in different groups was assessed by SA‐β‐gal staining. Scale bar: 50 μm. (C, D) Human NP cells untreated or pretreated with 20 μM Y27632 for 2 h prior to compression stress for 36 h were stained with MRTF‐A (green) and DAPI (blue) (n = 20). Scale bar: 10 μm. (E, F) mRNA levels of the ECM remodelling proteinases (MMP3, MMP13 and ADAMTS5) and ECM component (COL1A, COL2A and aggrecan) were measured after the human NP cells were treated withY27632. (G, H) The proportions of cells in each cycle were measured through flow cytometry in different groups. (I, J) CDK4, Cyclin D1, p21 and p53 expression levels in different groups were measured through Western blot analysis and normalized to that of GAPDH. Data were presented as the mean ± SD (n = 3). #No significance, vs compression group; *P < .05, vs compression group; **P < .01, vs compression group; ***P < .001, vs compression group

Furthermore, the percentage of SA‐gal‐positive cells was markedly decreased by Y27632 under compression stress (Figure 8A, B). Immunofluorescence analysis indicated that inhibition of the RhoA/ROCK1 pathway substantially inhibited the compression stress‐induced nuclear localization of MRTF‐A in human NP cells (Figure 8C, D). As expected, inhibition of the RhoA/ROCK1 pathway reduced ECM remodelling proteinases in human NP cells and reserved the fibrotic phenotype (Figure 8E, F). Moreover, flow cytometric analysis revealed that inhibition of the RhoA/ROCK1 pathway resulted in a decrease in the G1 phase and an increase in the S and G2 phase in human NP cells (Figure 8G, H). The expression levels of cell cycle related proteins, CDK4, cyclin D1, p21 and p53, also confirmed the same trend (Figure 8I, J). These results suggested that the RhoA/ROCK1 pathway activation mediated compression stress‐induced human NP cells senescence by regulating the actomyosin cytoskeleton remodelling.

## 4 DISCUSSION

**FIGURE 9:**
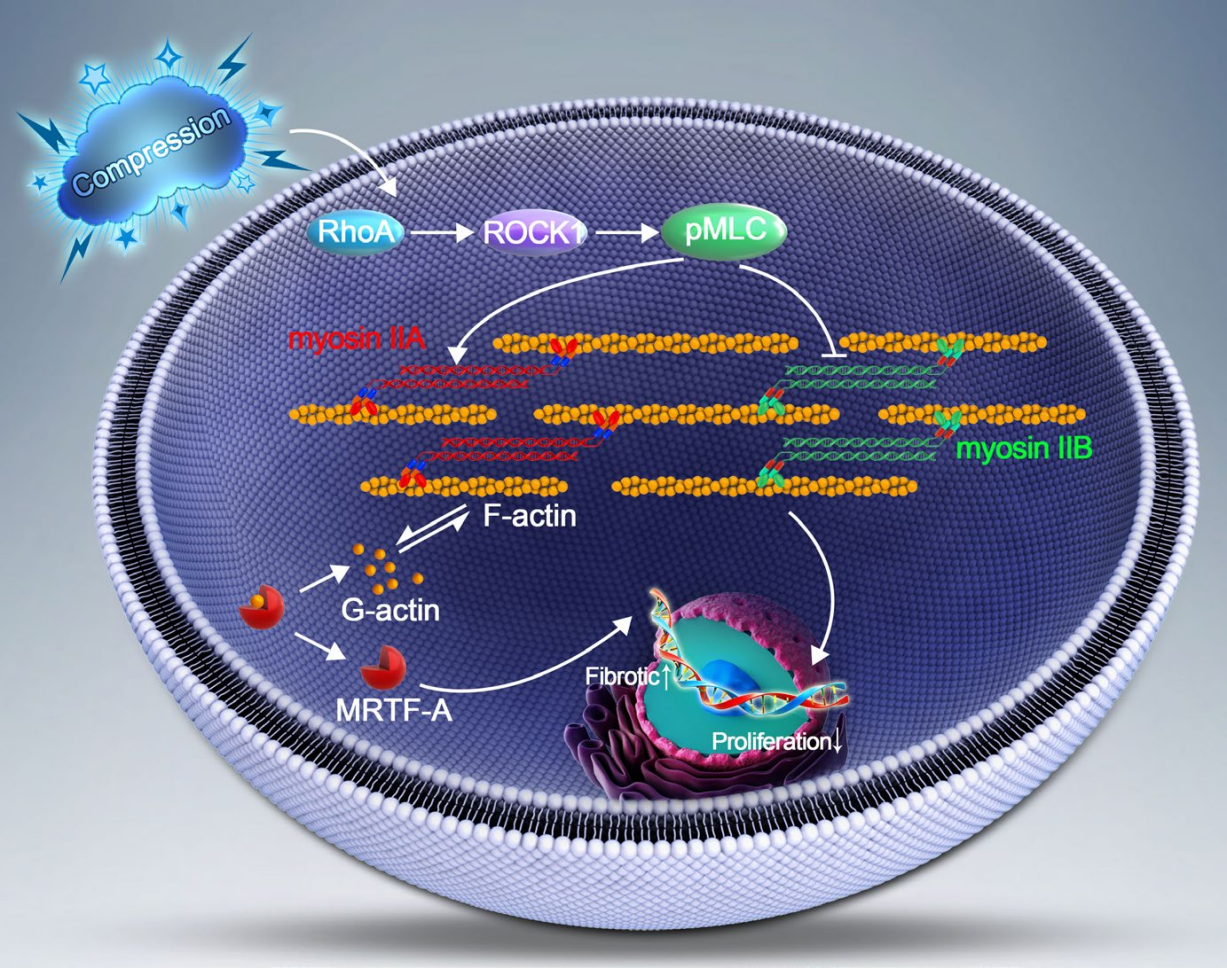
Schematic graph of the role of myosin IIA and IIB in compression stress‐induced senescence of NP cells. Compression stress induced the RhoA/ROCK1 pathway activation, which regulated the interaction of myosin IIA and IIB with actin. The actomyosin cytoskeleton remodelling was involved in the compression stress‐induced fibrotic phenotype mediated by MRTF‐A nuclear translocation and inhibition of proliferation in human NP cells

As the load‐bearing structure of the spine, the IVD is subjected to various mechanical stimuli in daily life.^35^, ^36^ Inappropriate or excessive compression applied to IVD contributes to IVD degeneration.^9^, ^37^ In our previous studies, we demonstrated that compression stress can induce apoptosis of NP cells and ECM degradation during IVD degeneration.^20^, ^21^ However, the roles of myosin II in human NP cells under compression stress is poorly understood. Here, we for the first time explored the roles of myosin II isoforms in the compression stress‐induced senescence of NP cells (Figure 9).

The actomyosin cytoskeleton is responsible for most force‐driven processes in cells and tissues.^14^, ^38^, ^39^ Although the actomyosin is comprised of the same major components, namely actin and myosin II, how these structures are assembled at the right time and place to maintain the normal physiological structure of the cell is an important question. Myosin II has been reported to form polarized cables to maintain tissue elasticity and cell shape upon mechanical stretch.^40^ Myosin polarity increased tissue stiffness to protect against fractures and injuries. Another study reported that myosin IIA forms bipolar filaments in red blood cells that are associated with F‐actin at the membrane.^41^ Myosin IIA activity regulated interactions with the spectrin‐actin network to control red blood cell biconcave shape and deformability. In our study, myosin IIA tended to be broadly in peripheral in normal human NP cells, while myosin IIB was distributed throughout the cytoplasm. Compression stress exposure induced dramatic changes in the reorganization of myosin IIA, IIB and F‐actin. Myosin IIA had a stronger association with actin filaments under compression stress than normal conditions. In contrast, compression stress reduced the interaction of myosin IIB and F‐actin. Overall, our results indicated that compression stress differentially regulated the interaction of myosin IIA and IIB with actin.

Cellular activity, such as growth and motility, is the result of a balance of forces. This balance is regulated by the coordinated operation of molecular motors.^42^, ^43^, ^44^ Disorder in the mechanical environment leads to disturbances in various cellular activities. It has been reported that myosin IIA drives cell retraction and maintains tensile adhesion, while myosin IIB drives outgrowth.^45^, ^46^ A recent study described the distinct roles of the myosin II submits in regulating actin cortex mechanics during cell division.^19^ While myosin IIA was mainly responsible for generating cortex tension, myosin IIB acted as a cortex stabilizer to maintain cortex tension. Myosin IIA depletion decreased cortex tension and slowed furrow ingression. In contrast, myosin IIA depletion increased intracellular pressure and drove faster cleavage furrow ingression. In our study, we firstly revealed that actomyosin cytoskeleton remodelling participated in the compression stress‐induced human NP cells senescence. Moreover, myosin IIA or IIB knockdown generated distinct effects under compression stress. Our results revealed that myosin IIA knockdown rescued the inhibition of proliferation, while myosin IIB knockdown further increased the expression of senescence‐related proteins. Cell division is a delicate process that requires a stable mechanical environment. We speculated that myosin IIA knockdown might provide the environment needed for cell division by reducing the intracellular forces; while myosin IIB exacerbated the imbalance of mechanical environment under compression stress. Thus, our data revealed the diverse effects of myosin IIA or IIB knockdown on compression stress‐induced human NP cells senescence.

The actomyosin cytoskeleton is involved in a dynamic cycle of polymerization and depolymerization. When cells are exposed to mechanical stimuli, monomeric G‐actin always aggregates into F‐actin to maintain tissue shape.^47^ Subsequently, MRTF‐A relaxes from its complex with G‐actin and is translocated into the nucleus where it associates with SRF to activate the transcription of target genes, notably fibroblast‐like matrix genes.^48^, ^49^ It has been reported that cells growth on a stiff matrix, representative of the pathologic stiffness of Crohn's strictures, expressed increased levels of fibrotic genes and was associated with nuclear localization of the transcriptional cofactor MRTF‐A.^50^ Another study demonstrated that mechanical strain induced the nuclear translocation of MRTF‐A and a pro‐fibrotic phenotype in human mitral valvular interstitial cells.^51^ A recent study revealed that NP cells in stiff substrate appeared spread and fibrotic shape opposed to the round shape in soft matrix.^52^ In addition, matrix stiffness increased nuclear translocation of MRTF‐A and induced a fibrotic phenotype in NP cells. The results of our present study showed upregulated nuclear expression and nuclear localization of MRTF‐A in human NP cells under compression stress. As expected, the inhibition of MRTF‐A nuclear translocation protected human NP cells against catabolism and preserved the primary phenotype under compression stress. However, the inhibition of MRTF‐A nuclear translocation did not rescue the compression stress‐induced decrease in proliferation of NP cells. Our data indicated that nuclear translocation of MRTF‐A mediated a fibrotic phenotype in human NP cells under compression stress while the inhibition of proliferation was independent of MRTF‐A.

The RhoA/ROCK pathway is known to act as a prominent player in mechanical signals and remodelling of the actomyosin cytoskeleton.^53^, ^54^ In addition, Rho/ROCK signals regulate the formation of lamellipodia and promote the degradation of ECM.^55^ It has been demonstrated previously that substrate stiffness regulated migration and invasion ability of adenoid cystic carcinoma cells via the RhoA/ROCK pathway.^56^ Another study also detected increased ROCK activity due to ECM rigidity; however, they found that ROCK1 and ROCK2 differentially regulated invadopodia activity through separate signalling pathways via contractile (myosin II) and non‐contractile (LIMK) mechanisms.^57^ Interestingly, curved microstructures were found to promote osteogenesis of mesenchymal stem cells by regulating the RhoA/ROCK pathway.^58^ In our study, we detected increased expression levels of p‐RhoA, ROCK1 and p‐MLC in human NP cells under compression stress and in the degenerative IVD tissues, while the expression of ROCK2 did not change. Furthermore, inhibition of the RhoA/ROCK1 pathway reversed the interaction of myosin IIA with actin and increased the interaction of myosin IIB with actin under compression stress. As expected, inhibition of the RhoA/ROCK1 pathway reduced the compression stress‐induced senescence and ECM remodelling in NP cells. Our results revealed that RhoA/ROCK1 pathway activation mediated compression stress‐induced human NP cells senescence by regulating the interaction of myosin IIA and IIB with actin.

In conclusion, we first demonstrated that compression stress differentially regulated the interaction of myosin IIA and IIB with actin in human NP cells. The actomyosin cytoskeleton remodelling was involved in the compression stress‐induced fibrotic phenotype mediated by MRTF‐A nuclear translocation and inhibition of proliferation in human NP cells. Moreover, our results indicated that RhoA/ROCK1 pathway activation mediated compression stress‐induced human NP cells senescence by regulating the interaction of myosin IIA and IIB with actin. It provided new insights into the development of therapy for effectively inhibiting IVD degeneration.

## CONFLICT OF INTEREST

The authors have declared that no competing interest exists.

## AUTHOR CONTRIBUTIONS

Cao Yang, Wencan Ke, Bingjin Wang and Wenbin Hua conceived and designed the study. Wencan Ke, Bingjin Wang, Wenbin Hua, Yu Song, Saideng Lu, Rongjin Luo, Gaocai Li and Kun Wang acquired, analysed and interpreted the data. Wencan Ke, Bingjin Wang, Wenbin Hua, Zhiwei Liao, Qian, Xiang and Shuai Li drafted and edited the manuscript. Wencan Ke, Bingjin Wang, Wenbin Hua, Xinghuo Wu, Yukun Zhang and Cao Yang critically revised the manuscript for intellectual content. All authors approved the final version of the manuscript.

## Data Availability

The data that support the findings of this study are available from the corresponding author upon reasonable request.
